# Validation of the Food Safe Zone questionnaire for families of individuals with Prader-Willi syndrome

**DOI:** 10.1186/s11689-024-09589-y

**Published:** 2025-02-08

**Authors:** Elisabeth M. Dykens, Elizabeth Roof, Hailee Hunt-Hawkins, Theresa V. Strong

**Affiliations:** 1 Department of Psychology and Human Development, Vanderbilt University, Nashville, TN USA; 2 Foundation for Prader-Willi Research, Covina, CA USA

**Keywords:** Prader-Willi syndrome, Hyperphagia, Clinical trials, PWS food safety

## Abstract

**Background:**

Prader-Willi syndrome (PWS), a genetic neurodevelopmental disorder, is characterized by hyperphagia and significant behavioral problems. Hyperphagic individuals with PWS are chronically hungry yet rarely feel sated, and often engage in food-seeking behaviors. To avoid life-threatening obesity in their children, families implement food security strategies (e.g., locking food sources, constant supervision around food, alerting others). Although widely used, these strategies have yet to be systematically examined. We thus developed and analyzed the psychometric properties of a new measure of these diverse strategies, the Food Safe Zone, and evaluated them in relation to hyperphagic symptoms and demographic variables. In doing so, we also shine a light on the extraordinary efforts of families in managing their children’s hyperphagia.

**Methods:**

Our team developed 20 FSZ items that were revised for clarity and completeness in an iterative feedback process with stakeholders, including parents, PWS specialists, and individuals with PWS. The FSZ was pilot tested, descriptive findings were reviewed by additional stakeholders, and then administered to 624 parents in a large-scale study. Based on an open-ended question, “Is there anything else you do to ensure food safety?” two additional items were added and evaluated in a follow-up study.

**Results:**

Principal component analyses revealed that 21 FSZ items loaded onto 5 factors that were readily interpretable, accounting for 67% of test variance: Alerting Others and Food Supervision in the Community; Locking or Restricting Food Sources; Checking for Food; At Home Supervision and Meals; and Avoiding Food Settings. Internal consistency and test-rest reliability were robust. Convergent validity analyses revealed that parents implemented FSZ strategies in response to the severity of their child’s hyperphagia, and not their child’s age, gender or PWS genetic subtype.

**Conclusions:**

The psychometrically sound FSZ holds promise for future research, especially on the effects of food safety tactics on family members. In future clinical trials, the FSZ could also be used to help parents think critically about their food safety tactics in relation to their child’s hyperphagia, or as an exploratory endpoint; if hyperphagia is lessened, so too may food safety tactics, thereby enhancing familial quality of life.

**Supplementary Information:**

The online version contains supplementary material available at 10.1186/s11689-024-09589-y.

Prader-Willi syndrome (PWS) is a neurodevelopmental disorder caused by the lack of paternally imprinted genetic information on chromosome 15q11-q13 [1]. Most cases (~ 70%) are attributed to paternal deletions in this region that vary in size, or maternal uniparental disomy (mUPD), when the child inherits two copies of the maternal chromosome 15 [2]. Behaviorally, people with PWS typically exhibit salient compulsivity, anxiety, needs for sameness, rigid thinking, hoarding non-food items, temper outbursts, repetitive questioning, and skin-picking [3, 4]. They also have mild to moderate deficits in intellectual and adaptive functioning [5], as well as in social cognition [6] and executive functioning [7, 8].

Hyperphagia, however, is the most striking and distinctive characteristic of PWS. Beginning in early childhood, hyperphagia is attributed to aberrant neural feedback mechanisms involved in appetite regulation and satiety [9]. As a result, hyperphagic individuals with PWS view the world through a lens of hunger; they are constantly hungry, yet rarely feel full or sated [10]. Their unrelenting hunger leads to food seeking behaviors, including sneaking food and manipulating others to obtain food. Hyperphagic severity and behaviors are often assessed with the informant-based Hyperphagia Questionnaire (HQ) [11] or an adapted version of the HQ, the HQ-Clinical Trials (HQ-CT) [12]. The HQ-CT has shown robust responsivity to treatment in previous clinical trials aimed at attenuating hyperphagia and related problems [13–15].

Hyperphagia in PWS is life-threatening as it can lead to medical complications associated with morbid obesity, gastric distention, and necrosis, and choking while binging or sneaking food [16, 17]. Risks of morbid obesity are lessened with growth hormone therapy (GHT), which is now a pediatric standard of care for treating growth hormone deficiencies in PWS. GHT is associated with increased linear height and lean muscle mass, and reduced body fat [18], as well as with significant advantages in cognition and adaptive behavior [19]. GHT does not, however, effectively treat or lessen the syndrome’s characteristic hyperphagia.

Fortunately, PWS has now garnered the attention of several pharmaceutical companies that are sponsoring clinical trials of novel agents aimed at attenuating hyperphgia and related symptoms in PWS [20]. Although these are promising developments, hyperphagia is currently best managed with environmental food safety strategies [2]. Common food safety strategies include locking food sources, supervising individuals whenever food is present, coordinating food intake with schools and other settings, and educating family members, friends, neighbors, teachers, and others about the need for food restrictions in PWS.

Many parents report that over time, their food safety practices become the “new normal” and an integral part of their daily routines. As one parent in our research program aptly observed, “Maintaining food safety has been automatic behavior 24/7 for 30 years.” If such practices become normalized, habitual or routine, they likely need less parental focused attention, concentration or cognitive effort to sustain, and may be associated with more efficient or less demanding meal preparation activities.

At the same time, accurate measurements of hyperphagia are critical both as an endpoint assessing treatment efficacy in clinical trials and as a criterion for trial eligibility. As parents typically serve as informants in clinical trials, it is important that they reconsider their food safety tactics, not in an automatic or routine way, but in a deliberate and reflective manner in relation to their child’s hyperphagia. Doing so is consistent with well-studied dual-process models of cognition [21].

In the present study, we developed and validated a novel index of food safety tactics in PWS, the Food Safe Zone (FSZ). Consistent with the FDA’s guidelines for Observer-Reported and Patient-Reported Outcomes [22], we developed the FSZ by involving multiple stakeholders and adhering to psychometric principles involved in questionnaire development. The study also analyzed the FSZ in relation to demographic variables (e.g., age, gender, PWS genetic subtype, parental socio-economic status). Beyond these analyses, we conducted analyzed parental comments to an open-ended question; “What did we miss? Are there other things that you do to ensure food security?”

In brief, this study aimed to establish the validity and reliability of the FSZ for future phenotypic and family research, and clinical trials. The FSZ could help parents think more critically about their food safety tactics prior to completing assessments of hyperphagia in future clinical trials. The FSZ could also be used as an exploratory endpoint in future trials; if hyperphagia is lessened, so too may food safety tactics, thereby enhancing familial quality of life. In this regard, the study also aims to shine a light on the extraordinary but under-recognized measures that parents use to manage the life-long challenges of their children’s hyperphagia.

## Methods

### FSZ item development and stakeholder input

Our research team developed a pool of 20 items that tapped common, readily observable food safety practices. Items were gleaned from interviews, data, and clinical notes from over 325 families enrolled in our previous and current PWS research programs.

Parents of 5 individuals with PWS provided initial feedback on the clarity of items, the proposed response scale and if additional items were needed to measure food safety, i.e., construct validity. Parents suggested a rewording of 8 items and nominated 5 additional items on the frequency that individuals with PWS attend events that do or do not involve food, with or without parental supervision.

The revised, 25-item FSZ draft was then vetted by 12 parents of individuals with PWS, aged 5 to 33 years, in 4 different, 40-minute focus group sessions held via Zoom. Parents concurred that the wording of the FSZ was clear and did not suggest additional items. Instead, parents specified how they implemented various FSZ strategies, and expressed appreciation for the opportunity to talk about food safety with others.

Finally, we solicited input on the FSZ via a Zoom focus group with 5 adults with PWS, aged 20 to 42 years. Group members were forthright about their struggles with hunger and food-seeking and did not suggest additional items. They also emphasized the importance and helpfulness of securing food, educating others about PWS, and maintaining a predictable meal and snack schedule.

### Pilot testing and additional stakeholder input

The revised, 25-item FSZ was pilot tested in 133 parents of individuals with PWS aged 5–43 years (see Participants, Table 1). Respondents were asked to complete the questionnaire based on the last month, using the following rating scale: 1 = Never/Rarely; 2 = Some of the time; 3 = Most of the time; 4 = All the time. The FSZ also included an open-ended question, “What did we miss? Are there other things you do to ensure food security?”

**Table 1:** Demographics of participants with PWS and their families for the pilot, large-scale and follow-up studies

	Pilot	Large-Scale	Follow-up
Demographic Variables	M (SD) or %	M (SD) or %	M (SD) or %
N	133	624^a^	119
Age, Years	18.86 (7.89) Range: 5–43	18.60 (10.65) Range: 5–59	15.25 (7.75) Range: 5–44
Gender	42.1% Male 57.9% Female	46% Male 54% Female	48% Male 52% Female
**Genetic Subtypes**			
Paternal Deletion	61.1%	50.8%	48.8%
mUPD	32.8%	34.4%	43.1%
Imprinting Deficit	3.8%	3.2%	3.1%
Other	2.3%	2.3%	1.3%
Unknown		9.3%	3.7%
**Education** **Maternal/Paternal**			
High School 2-year college 4-year college Post-graduate	21.4%, 27.0% 16.8%, 10.3% 31.3%, 32.5% 30.5%, 30.2%	17.2%, 24.4% 29.8%, 30.6% 34.2%, 26.9% 18.9%, 18.1%	18.8%, 23.3% 15.0%, 11.3% 28.1%, 31.4% 38.1%, 34.0%
**Annual Income**			
$10,000 - $30,000 $31,000 - $50,000 $51,000 - $70,000 $71,000–100,000 > $100,000 Unknown	6.1% 4.5% 13.8% 20.8% 54.6% --	5.8% 16.7% 16.0% 17.1% 35.3% 9.1%	8.8% 8.8% 13.8% 16.4% 52.2% ---
**Race**,** Ethnicity**			
White	92.3%	85.2%	88.7%
Hispanic	3.1%	7.4%	5.2%
Asian	2.3%	2.5%	3.5%
Black	2.3%	2.3%	2.6%
Multi-racial		2.6%	

^a^Large scale study included 133 pilot and 491 participants from FPWR’s registry

Descriptive pilot data (e.g., means, frequencies) were then reviewed by members of FPWR’s Clinical Trials Consortium, specifically 3 parents and 5 PWS professionals. Consortium members concurred that the FSZ thoroughly assessed common food safety practices and that items were clearly worded.

### Large-scale administration

The FSZ was administered to additional parents of individuals with PWS via The Global PWS Patient Registry, a secure, web-based Registry sponsored by the Foundation for Prader-Willi Research (FPWR) and hosted on the National Organization for Rare Disorders “IAMRARE” registry platform [23]. Registrants are asked to complete medical and behavioral questionnaires every six months, a time frame established by FPWR to reduce parental burden of more frequent assessments. Questionnaires included in the Registry change in response to the needs of researchers, clinicians and clinical trial sponsors, making it difficult to determine if there are differences in registrants who did or did not respond to the FSZ. The Registry garnered 491 respondents, and 304 (62%) completed the FSZ 6 months later.

### Respondent feedback and follow-up study

A follow-up study was conducted to assess two additional items gleaned from parental responses to the open-ended question, answered by 75% of participants. Our team reviewed these responses to determine if they represented novel strategies not included in the FSZ or were instead descriptions of how families enacted the tactics already included in the FSZ (see Results). Based on this review, two additional questions were added: “Making a food plan for child prior to attending events, outings, restaurants,” and “Avoid eating in front of child unless they are also eating. We added these two items to the FSZ and evaluated them in a follow-up study of 119 parents.

### Informed consent and IRB approval

Approval for this study was obtained by the Vanderbilt University Institutional Review Board, Integrated Science Committee. Vanderbilt participants provided written, informed consent using the e-consent function of RedCap, a secure, web-based data collection platform [24]. After consenting, parents were invited to complete the FSZ, HQ-CT and Demographic questionnaires on RedCap. Additional study approval was obtained for participants recruited from the FPWR Patient Registry. Prior to collecting data from the Registry, the study was reviewed and approved by FPWR’s research committee and IRB. All registrants in FPWR’s Patient Registry gave approval for their de-identified data to be used for research purposes.

## Participants

As hyperphagia typically onsets between 4 ½ to 8 years [25], the study included parents of individuals PWS aged 5 years through adulthood. Pilot study participants (*n* = 133) were recruited by our team at Vanderbilt University via postings on PWS-related social media platforms, and announcements at PWS conferences. As previously described, participants for the large-scale study (*n* = 491) were recruited via the FPWR Patient Registry.

Table 1 summarizes demographic variables for participants in the pilot and large-scale studies. No significant differences emerged between these two recruitment sources in participant demographics or FSZ scores. As such, they were combined to form a large group (*n* = 624) to increase power and facilitate statistical analyses.

Participants in the follow-up study (*n* = 119) were recruited from both Vanderbilt University and FPWR (see Table 1). After consenting, they completed the 27-item FSZ, which included the two additional items generated by parent feedback. Approximately 15% of those in the follow-up study had completed the 25-item FSZ one to two years prior either in Vanderbilt’s Pilot study, or FPWR’s Patient Registry.

Across the pilot, large-scale and follow-up studies, participants ranged in age from 5 to 59 years, with a mean age of 17.60 years. As Table 1 depicts, the majority of participants had genetic subtype confirmation of their PWS diagnosis, while just 62 participants had clinical diagnoses of PWS with unknown genetic subtypes.

### Other measures

#### Demographics

Parents completed a demographic form asking for the age, gender, race, and genetic subtype of their individual with PWS. Parental education and annual family income were also obtained. Body Mass Indices (BMI’s) were not consistently acquired across data sources. Given food-safety practices, however, BMI’s do not necessarily map onto hyperphagic severity; for example, one can have a normal BMI and still exhibit significant food-seeking and other hyperphagic symptoms [4, 10]. Thus, hyperphagia is typically used as an outcome measure in clinical trials [20].

#### Hyperphagia questionnaire-clinical trial

The HQ-CT was administered to all participants to determine the convergent validity of the FSZ [12]. This 9-item, informant-based questionnaire assesses two components of hyperphagia in PWS; hyperphagic drive or severity, and self-directed food seeking behaviors using a 0 to 4 scale. It has been used as an endpoint assessing treatment efficacy in clinical trials [13–15].

## Statistical analyses

### Factor analyses

Using the combined dataset (*n* = 624), principal component analyses (PCAs) were conducted to identify the latent factor structure of the FSZ. Separate PCA’s were performed using orthogonal (i.e., varimax) versus oblique rotations (i.e., equimax) to assess which rotation yielded the most readily interpretable, conceptually meaningful solutions [26]. Final analyses used the orthogonal solution.

PCAs adhered to well-established criteria [26]. These included Kaiser’s criteria with an eigenvalue > 1 and inspecting the Scree Plot to confirm the number of factors. We also ensured that items loading onto factors had a common conceptual meaning, with nominal cross-loading across factors, and that factor loadings and communalities were > 0.40, Bartlett’s Test of sphericity was significant, and the Kaiser-Meyer-Olkin measure of sampling adequacy was close to 1.

Although PCAs were conducted in the follow-up study, the sample size for these analyses was relatively small (*n* = 119), falling below conventional rules of thumb in factor analyses [27]. As such, the goals of these analyses were limited in scope. Specifically, we assessed if the two new items loaded onto factors that made conceptual sense, and if their inclusion compromised the overall structure of the FSZ as established in the large-scale study.

### Internal consistency

Cronbach’s alphas determined the internal consistency of items within each FSZ factor, and for the overall FSZ in both the large-scale and follow-up studies.

### Test-retest reliability

Test-retest reliability was assessed in 304 participants from the large-scale study who completed the FSZ at time 1 and again 6 months later. To minimize test-retest measurement error, we ensured that raters were the same across assessments. We first compared FSZ scores between Time 1 and Time 2 in matched t-tests. Intraclass Correlation Coefficients (ICCs) were then calculated, which incorporate both the degrees of agreement and correlations between Time 1 and Time 2 [28]. ICCs were based on a single measurement and absolute agreement in a two-way, mixed-effects model in which participant effects were randomized and measure effects were fixed.

### Demographic analyses

Pearson correlations, t-tests or Chi-Squares assessed relations between the FSZ total or factor scores with PWS genetic subtypes, gender, parental education or income, and age. Age was also assessed by dividing participants into three, developmentally appropriate age groups; children (5–12 years), adolescents (13–19 years) and adults (20–59 years; see Results).

### Convergent validity

As food safety tactics are implemented in response to hyperphagia, we predicted that scores on the HQ-CT would be positively associated with the FSZ. Pearson correlations were calculated between the HQ-CT and FSZ total and factor scores. Linear regressions were then conducted with FSZ factors as predictors of the total HQ-CT. To further assess relations between the FSZ and hyperphagia, the sample was divided into tertiles based on their total HQ-CT score. ANOVAs then compared FSZ raw factor scores across participants who were in the lowest, middle, and highest tertile HQ-CT groups.

### Open-ended question analyses

A full 75% of parents responded to the open-ended question. These comments offer novel insights into how parents implement food-safety practices that complement and extend formal statistical analyses of the FSZ. The team thus reviewed these comments without using predetermined categories and together observed that they fell into straightforward content categories. Most comments expanded on how parents specifically implemented FSZ strategies (See Results).

## Results

### Factor analyses

#### Large-scale study

Preliminary PCAs with the combined dataset revealed that 6 items failed to load onto any factor. Five items dealt with allowing individuals with PWS to attend events with or without food present or parental supervision. These items were infrequently endorsed (13–25%), most likely due to confounds regarding the need for supervision related hyperphagia and/or cognitive disabilities. The 6^th^ item, “weight child at least weekly” was also infrequently endorsed (18%). In retrospect this item is not a measure of food security but is instead a down-stream indicator of consumed food.

The final PCA with 19 items yielded five factors that collectively accounted for 67.02% of test variance. A common conceptual meaning could be readily applied to these factors, specifically: Alerting Others and Food Supervision in the Community (4 items, 15.32% of rotated variance); Locking and Restricting Food Sources (6 items, 15.21% of variance); Checking for Food (3 items, 14.62% of variance); At Home Supervision and Meals (4 items, 11.80% of variance); and Avoiding Food Settings (2 items, 10.07% of variance).

The Avoiding Food Settings factor contained two items instead of the conventional three or more items. As such, and as recommended Worthington and Whittaker [29], we ensured that these 2 FSZ items were strongly correlated (*r* = .72), shared a conceptual meaning, and were relatively unrelated to other FSZ items (r’s ranged from .12 to .25).

Table 2 presents the FSZ items that loaded onto these 5 factors, their factor loadings, and communalities. All factor loadings were acceptable (> 0.40), yet more stringent guidelines [30] suggest that 17 items had either excellent (> 0.71) or very good (> 0.63) factor loadings, and just 2 items were deemed fair to good (0.40–0.45). Similarly, communalities indicated that all items were valuable in contributing to the test variance of their respective factors. Table 2 also includes the percentages of parents who endorsed each item as all or most of the time versus sometimes or never/rarely.

**Table 2:** FSZ items that loaded onto 5 factors, factor loadings, communalities, item means and relative frequencies from the large-scale-study

FSZ Factor Labels and Items	Factor Loading	Communalities	Item Means (SD)	All or Most of the Time	Some or None of the Time
**1. Alert Others, Supervision in the Community**					
Make sure adults involved with my child are aware of his/her food issues.	.79	.66	3.68 (.74)	91.7%	8.3%
Alert others of child’s food issues (to ensure they do not give child access to food).	.78	.67	3.47 (.91)	85.0%	15.0%
Make sure they are supervised while away from home.	.76	.66	3.64 (.75)	89.7%	10.3%
Ensure child has no access to other people’s food at school, camp, or work.	.67	.53	3.06 (1.16)	72.1%	27.9%
Make a food plan for child prior to attending events, outings, restaurants^a^	.62	.56	3.24 (.89)	79.7%	20.3%
**2. Lock, Restrict Food Sources**					
Lock up pantry or cabinets where food is kept	.90	.87	2.64 (1.35)	55.3%	44.7%
Lock up refrigerator or freezer	.89	.80	2.46 (1.40)	49.9%	50.1%
Ensure there is no food left on counter tops, tables, or other areas of access	.65	.68	2.99 (1.35)	70.2%	29.8%
Lock up trash, compost, or recycling bins	.50	.51	1.62 (1.35)	20.5%	79.5%
Keep money or credit cards from child	.46	.50	2.32 (1.36)	46.0%	54.0%
Use security features (alarm, camera) in home to monitor food access	.40	.47	1.62 (1.23)	20.7%	79.3%
**3. Check for Food**					
Check their person (pockets, pat down, shoes, etc.) for food, wrappers, or money	.87	.82	1.70 (.94)	18.6%	81.4%
Check their belongings or bedroom for food, wrappers, or money	.87	.83	1.98 (1.03)	28.0%	72.0%
Check on child in bathroom to be sure he or she is not eating food	.84	.75	1.69 (.99)	19.6%	80.4%
**4. At Home Supervision, Meals**					
Make sure child is busy while at home	.72	.66	2.86 (.85)	67.6%	32.4%
Avoid eating in front of child unless they are also eating^a^	.69	.55	2.86 (1.02)	67.0%	33.0%
Aware of where child is at home all the time (but may not be in sight of caregiver)	.63	.63	3.39 (.86)	85.6%	14.4%
Supervise - always have eyes on at home	.61	.62	3.02 (.93)	73.9%	26.1%
Make sure that meals are planned and on time	.50	.41	3.05 (.81)	79.4%	20.6%
**5. Avoid Food Settings**					
Avoid taking child to the grocery store	.85	.79	2.08 (1.07)	34.3%	65.7%
Avoid taking child to restaurants	.84	.80	2.18 (1.02)	34.3%	60.2%

^a^items are from the follow-up study and included here for reader convenience. See Additional Table 1 Table for the complete PCA results from the follow-up study

#### Follow-up study

PCA’s conducted in the follow-up study assessed the impact of the 2 additional items on the overall structure of the FSZ. The PCA with varimax rotation yielded 5 factors that collectively accounted for 62.75% of test variance. These 5 factors recapitulated the factors derived in the large-scale study, albeit with slightly different factor loadings, communalities, and frequencies (see Additional File Table 1 for these results). Importantly, the two new items loaded onto factors that were readily interpretable. The item “Make a food plan for child prior to attending events, outings, restaurants” loaded onto the Alerting Others and Supervision in the Community factor, and “Avoid eating in front of child unless they are also eating” was consistent with other tactics families implement in the At Home Supervision and Meals factor. These two items were frequently endorsed, 79.7% and 67.0%, respectively. T-tests compared FSZ factor mean scores between the large-scale and follow-up studies, none were significant (see Additional File Table 2 for these results).

### Internal consistency

Cronbach’s alphas were calculated for each of the five factors derived from the large-scale and follow-up studies. Conventional rules of thumb suggest that alphas > .70 and < .90 are considered good [31]. As summarized in Table 3, all alphas fell into this range and varied from .73 to .89.

**Table 3:** Cronbach alphas for FSZ factors in both the large-scale and follow-up studies

FSZ Factors	Cronbach Alpha’s
Alert Others, Supervise in Community Large-Scale Study	.79
Alert Others, Supervise in Community Follow-up Study	.76
Lock, Restrict Food Sources Large-Scale Study	.82
Lock, Restrict Food Sources Follow-up Study	.79
Check for Food Large Scale Study	.89
Check for Food Follow-Up Study	.89
At Home Supervision, Meals Large-Scale Study	.76
At Home Supervision, Meals Follow-Up Study	.73
Avoid Food Settings Large-Scale Study	.81
Avoid Food Settings Follow-Up Study	.74
Total FSZ Raw Scores Large Scale Study	.89
Total FSZ Raw Scores Follow-Up Study	.86

### Test-retest reliability

Matched t-tests conducted with the total FSZ scores between Time 1 and Time 2 proved nonsignificant, Time 1 *M* = 51.93, *SD* = 13.07, Time 2 *M* = 51.62, *SD* = 12.65. Intraclass correlations and their corresponding 95% confidence intervals are presented in Table 4. Based on conventional criteria, ICCs were all in the good range [28].

**Table 4:** FSZ time 1 to time 2 intraclass correlations and 95% confidence intervals

FSZ Factors	Intraclass Correlations (95% CI)
Alert Others, Supervision in the Community	.74 (.67–.78)
Lock, Restrict Food Sources	.88 (.86–.91)
Check for Food	.78 (.74–.82)
At Home Supervision, Meals	.75 (.69–.79)
Avoid Food Settings	.65 (.58–.71)
Total FSZ	.88 (.85–.90)

### Combined large-scale and follow-up studies

Separate analyses of the large-scale and follow-up studies yielded the same factor structure, as well as similar mean FSZ scores and Cronbach’s alphas. These datasets were thus combined (*n* = 743) for subsequent analyses of relations between the FSZ and demographic variables, and of the convergent validity of the FSZ with the HQ-CT. Data were missing from 6 participants for some analyses.

### FSZ and demographics

ANOVAS or t-tests revealed no significant differences in FSZ total or factor scores across gender, PWS genetic subtypes, race, and parental income or education. The correlation between age and total FSZ scores was relatively small, *r* (735) = 0.19, *p* < .001. Correlations also assessed relations between age and the HQ-CT’s total score, and the HQ-CT’s severity/drive and food-seeking behavior domains. Only the self-directed food-seeking behavior domain proved significant, *r* (735) = 0.22, *p* < .001.

To further explore these findings, participants were divided into three developmentally appropriate age groups: children aged 5 through 12 years (*n* = 268; *M* = 8.31 years, *SD* = 2.44); adolescents aged 13–19 years (*n* = 247, *M* = 16.16 years, *SD* = 2.40) and adults aged 20–59 years (*n* = 228, *M* = 30.50 years, *SD* = 8.36). Between-age group ANOVAs were then conducted with both FSZ and HQ-CT.

As summarized in Table 5, significant age group differences were found in 4 FSZ factors. Bonferroni post-hocs revealed that locking food sources differed significantly between all age groups, and that the adolescent and adult groups scored higher than children in checking for food and avoiding food setting. Children, however, scored higher than the 2 older age groups in the alerting others factor. Effect sizes (η^2^) were large for the locking factor, medium for checking for food, and small for the remaining two factors.

**Table 5:** FSZ and HQ-CT mean raw scores, and F, p and η^2^ values across three age groups

FSZ Factors	5 to 12 M (SD)	13 to 19 M (SD)	20 to 59 (M (SD)	F, p	η^2^
N	268	247	228		
Age	8.31 (2.44)	16.16 2.40)	30.50 8.36		
Alert Others, Community Supervision	15.12^A^ (3.14)	14.21^B^ (3.27)	13.95^B^ (3.00)	9.63***	.025
Lock, Restrict Food Sources	11.62^A^ (4.71)	13.58^B^ (5.43)	16.22^C^ (5.00)	58.74***	.138
Check for Food	4.42^A^ (2.30)	5.53^B^ (2.74)	5.91^B^ (2.71)	22.24***	.057
At Home Supervision, Meals	12.67 (3.00)	12.73 (3.05)	12.80 (2.64)	.177	NS
Avoid Food Settings	3.77^A^ (1.76)	4.37^B^ (1.91)	4.43^B^ (1.89)	9.88***	.026
Total FSZ	47.60^A^ (10.40)	50.42^B^ (12.03)	53.36^C^ (10.94)	30.74***	.063
**HQ-CT**					
Hyperphagic Drive, Severity	12.77 (5.32)	13.96 (6.12)	13.13 (5.58)	2.59	NS
Food-Seeking Behaviors	4.06^A^ (2.84)	4.63^B^ (3.22)	5.03^C^ (3.19)	6.47***	.017
Total HQ-CT	16.83 (7.31)	18.59 (8.55)	18.16 (7.96)	3.14*	.009

***p<.001; *p< .05. *NS *Nonsignificant. Different superscript letters depict significant post-hoc group differences

As shown in Table 5 the ANOVA assessing age group differences in the total HQ-CT was marginally significant. Consistent with correlational analyses, this finding is driven by the more robust age group differences in the HQ-CT’s food seeking domain.

### Convergent validity

As expected, total FSZ and HQ-CT scores were positively correlated *r* (735) = 0.54, *p* < .001. Indeed, total HQ-CT scores were significantly correlated with all 5 FSZ factors (r’s range = .29 to .50, *p*’s < .001).

The regression assessing FSZ factors as predictors of the total HQ-CT was significant, *F* (5,717) = 66.13, *p* < .001, adjusted R^2^ = 0.324. Four FSZ factors were significant predictors (*p*’s < 0.001), including Checking for Food, *t* = 11.19, β = 0.401; Locking Food Sources, *t* = 6.30, β = 0.256; and At Home Supervision and Meals, *t* = 3.21, β = 0.153. Avoiding Food Settings was marginally significant, *t* = 2.33, *p* = .020, β = 0.082.

Probing deeper, participants were categorized into tertiles based on their total HQ-CT raw score; ANOVAs then compared FSZ scores across hyperphagia tertiles. The lowest tertile included 238 participants (32%), the middle tertile contained 245 individuals (33%) and the highest group had 260 participants (35%). Because the tertile groups did not significantly differ in age, age was not controlled for in ANOVAs. As summarized in Table 6, significant differences were found between tertiles and all five of the FSZ raw factor scores. Bonferroni post-hocs revealed that all groups differed significantly from one another, with one exception. The medium and highest tertiles had comparable scores on the At Home Supervision and Meals factor. Effect sizes (η^2^) were large for 3 factors, and medium to large for 2 factors.

**Table 6:** Comparisons of FSZ raw factor scores across tertiles of participants with low, medium, and high total HQ-CT scores

FSZ Factors	Low HQ-CT M (SD)	Medium HQ-CT M SD)	High HQ-CT M (SD)	F, p	η^2^
N	238	245	260		
HQ-CT Total	9.62 (1.67)	15.78 (2.10)	26.73 (5.50)		
Age	17.30 (11.47)	17.76 (10.93)	18.27 (9.16)	.561	NS
Alert Others, Community Supervision	13.24 (3.94)^A^	14.82 (2.74)^B^	15.20 (2.74)^B^	26.72***	.069
Lock, Restrict Food Sources	10.23 (4.81)^A^	13.72 (5.14)^B^	16.24 (4.60)^C^	91.87***	.204
Check for Food	3.88 (1.61)^A^	5.00 (2.42)^B^	6.65 (2.94)^C^	79.79***	**.**182
At Home Supervision, Meals	11.05 (2.25)^A^	13.09 (2.52)^B^	13.88 (2.22)^C^	70.05***	.163
Avoid Food Settings	3.37 (1.87)^A^	4.20 (1.80)^B^	4.82 (1.68)^C^	39.11***	.098

***p< .001. *NS *Nonsignificant. Different superscript letters depict significant post-hoc group differences

### Analyses of open-ended question responses

Four sets of findings emerged from the open-ended question. First, 21 respondents indicated that their individual with PWS did not engage in food-seeking behaviors. The majority of these (*n* = 16, 76.2%) were noted by their parents to have a medical or psychiatric exceptionality that impacted their food intake. These exceptionalities were: severe developmental delays, including four, minimally verbal individuals with autism spectrum disorder, and two individuals with persistent psychotic episodes. The remainder had such medical complications as Type I diabetes (*n* = 3), multiple food allergies with anaphylaxis (*n* = 3), severe hypothermia (*n* = 1), overwhelming fatigue (*n* = 1), being G-Tube dependent (*n* = 1) and being paralyzed and in a wheelchair (*n* = 1). We were curious if eliminating these individuals would substantially shift PCA or other analyses; it did not.

Second, parents overwhelmingly responded to the open-ended question by elaborating on the specific ways that they individualized or implemented FSZ tactics. As previously described, our team reviewed these descriptions without predetermined categories and readily classified them into 6 categories that dovetailed with FSZ items. Parental remarks were straightforward and did not require judgment calls, allowing the team to place them logically and with full agreement into the following categories: locking and securing food sources; scheduling meals and snacks; managing restaurants, parties, and family gatherings; eating and discarding food at home; and working with schools. Table 7 summarizes examples of parental responses within these categories, as well as the frequencies of them relative to the number of respondents to the open-ended question. As parents could elaborate on several strategies, the frequencies noted in Table 7 exceeds 100%.

**Table 7:** Examples and frequencies of parental descriptions of food FSZ tactics derived from the open-ended question

**Locking and Securing Food Sources (37%**^**a**^)
∙We modified our kitchen/living area by creating two separate rooms and then installed locking doors. ∙We moved the entire kitchen to the basement with a locked, alarmed door and unique key. ∙Instead of locking cabinets or fridge, we lock off entire kitchen with a metal gate. ∙We turned an unused bedroom into a pantry with a self-closing door and pin code electronic lock. ∙We use motion sensors, open/close sensors, cameras, and locks. If he gets food, the cameras tell us “how.” ∙Our keys to kitchen cabinets are locked in a safe. ∙I have a wind chime attached to my fridge door with a magnet, to alert me when it is opened. ∙We lock up purses or backpacks where we might have medicine, gum, or mints. ∙Must keep money, food, and snacks from being left in cars, just in case, we always lock the car. ∙Bathroom cupboard locked so no access to toothpaste, mouthwash, flavored antacids, cough drops, or vitamins. ∙We also need to lock up our liquor cabinet.
**Preparing, Eating and Discarding Food At Home (25.6%)**
∙Proportion food as soon as it is brought home instead of leaving it in bulk packaging. ∙Ensure that only foods that she can eat comes into the house. No junk food allowed. ∙I purchase less food, which means going to the market more often. ∙EVERYONE eats at the table. No food allowed in bedrooms, while watching TV, etc. ∙Scrape leftover food into the garage disposal and run the garbage disposal frequently to ensure no food remains. ∙Must discard bones from chicken, ribs, etc. where he cannot get to them. ∙Table and floor are cleaned after every meal, otherwise he would eat dropped food or lick the floor. ∙Siblings must keep treats or forbidden food a secret, store them in their locked bedrooms, and immediately ∙discard wrappings outside in trash. ∙Don’t allow siblings or family members to keep food in their rooms, desks, or private spaces. Only diet drinks. ∙I sleep on the sofa to supervise and prevent night food seeking. ∙I am always within an arm's reach of her because she is an elopement risk, so I even sleep with her
**Alerting Others and Supervision in the Community (14.3%)**
∙When he uses Lyft or Uber, I get a link to follow the route to ensure no food-related extra stops. ∙We live nearby restaurants, so we gave them a picture of our son and a caption: Please don't feed me. Call immediately if I am alone. And our phone numbers. ∙The police are aware of her disorder as she ran away to steal food. ∙All my neighbors know about my daughter’s condition, and to call me is she asks for or steals food. ∙Must monitor church coffee hours, candy dishes at the bank, doctor’s office, etc. ∙Alert everyone! Van drivers, Special Olympics coaches, neighbors, church groups. ∙I let people in charge at her day program know about her food issues - then I must trust their supervision. ∙She attends classes at a vocational school and must text us a picture of her lunch tray.
**Working with Schools (13.2%)**
∙Written into his IEP that food cannot be used in lessons or offered as a reward. ∙We lock her lunch box through the zippers. She hands it over to the school bus driver. ∙Try to ensure a food free curriculum at school, including lessons with pictures of food. ∙Needed to get a 1:1 aide at school to supervise food. ∙She buys lunch at school once a week. We choose healthy options from the menu; staff ensures her tray is okay. ∙Her lunch/snacks are out of sight. We use colored containers, so staff recognize if she is eating other food. ∙We keep her from attending school parties or celebrations that involve food or treats. ∙We have the teacher bring her lunch to eat in her classroom. This avoids her going to the cafeteria.
**Scheduled Meals, Snacks (17.3%)**
∙Maintain regular times for breakfast, snack, lunch, snack, dinner, and dessert. ∙Post menus each day. Measure food so she doesn’t manipulate different caregivers into giving her more food. ∙Schedule, schedule, and schedule. And always have a back-up plan! ∙Keeping a consistent schedule works the best, it reduces uncertainty or anxiety about next "snack" or "meal." ∙She gets the same number of meals/snacks, the same number of calories, at the same time every day. ∙I always pack a snack in my purse if we are stuck in traffic or running behind schedule.
**Restaurants, Parties, Family Gatherings (20.2%)**
∙Never let him go to bathroom alone at restaurants, he will grab food from other tables that was left behind. ∙Must hold hands walking through a restaurant as she will take food off plates. ∙Must stay with me or an attendant in any store the entire time as she is very fast getting food. ∙Take waitstaff aside and educate them about PWS and why we will order for her. ∙Remove all condiments from table in a restaurant. ∙Preview restaurant menu and pick two options before going out to a restaurant. ∙She must take half of her restaurant meal in a to-go bag for the next day. ∙We sit next to him at family parties to ensure appropriate portions. ∙We don’t attend family gatherings, it’s too stressful for everyone. ∙Avoid buffet style parties or restaurants!! ∙At parties we give him a plate of food and tell others not to feed him. But this is awkward and hard to monitor

^a^Percentages are based on the number of respondents to the open-ended question

Third, 9.1% of respondents offered ways that they help their individual with PWS to eat. Although not necessarily food security tactics, parents noted that they used smaller plates so that food quantities seem larger, pre-plated meals, cut-up portions to avoid overstuffing, and encouraged their child to put down their fork after every few mouthfuls. Several participants also emphasized the importance of teaching their individual with PWS about proper nutrition and establishing healthy eating habits in all family members.

Finally, 5 respondents offered that their individual with PWS consumed non-edible items (e.g., pieces of a TV remote, hair). Some people with PWS may eat unpalatable items (e.g., stick of butter, frozen meat, garbage, pet food), or endorse a willingness to eat unusual food combinations [32]. However, pica, or eating non-food items, has not been widely reported in the PWS literature.

## Discussion

The FSZ emerged as a psychometrically sound index of the tactics that parents use to manage their child’s hyperphagia. Beyond the psychometrics of the FSZ, findings are discussed in relation to hyperphagia, age, and how the FSZ may be used in future research or clinical trials aimed at attenuating hyperphagia. We also review how parents tailor their food safety tactics to meet the individual needs of their child with PWS, and the impact of doing so on their well-being.

Consistent with best practices in creating novel questionnaires [22], the FSZ was developed with input from multiple stakeholders, including parents, PWS specialists and researchers, and individuals with PWS. In an iterative feedback process, items were added, or revised for clarity, and then pilot-tested, revised, and administered to parents in a large-scale study. Based on parental responses to an open-ended question, two additional items were added, and subsequently evaluated in a follow-up study. This multi-step process helped ensure both the construct and content validity of the FSZ.

Other psychometric properties of the FSZ were also robust. PCAs in the large-scale study yielded five, conceptually meaningful factors that collectively accounted for 67% of test variance. Communalities indicated that all items contributed meaningfully to their respective factors. Cronbach’s alphas revealed strong internal consistency of items within each factor, and for FSZ total scores. As well, ICC’s suggest strong test-retest reliability. Indeed, mean FSZ scores at Time 1 and 2 were almost identical, suggesting relatively stability in FSZ tactics over this 6-month time interval.

Importantly, analyses in the follow-up study, with two additional items, revealed the same overall factor structure as the large-scale study, albeit with slight differences in factor loadings, communalities, and frequencies. The new items were frequently endorsed, loaded onto factors that made conceptual sense, and no differences in mean FSZ scores were found between the large-scale and follow-up studies. Taken together, findings justify the use of the final, 21-item version of the FSZ questionnaire in future research or clinical trials. Such future work could either use the sum of the raw scores across all five factors, or from selected factors that align with research hypotheses.

Age was the only demographic variable significantly associated with the FSZ. Relative to children, parents of adolescents and adults were more apt to lock food sources, check for food and avoid food settings. Parents of children, however, scored higher than their counterparts in the alerting others about food issues. These findings are best understood in relation to significant age-related increases in the HQ-CT’s food-seeking behavior domain, even as hyperphagic severity or drive remained relatively stable in participants.

With advancing age and development, people with PWS may become more skilled or adept in finding or sneaking food. Indeed, they are known to exhibit such ingenious food-seeking strategies as unscrewing hinges to kitchen cabinets at night, dangling food on strings in the heating vents, and memorizing credit card numbers and ordering food deliveries to a friend’s address. Such tactics require foresight and planning yet contradict well-documented deficits in executive functioning in people with PWS, especially task-switching and planning abilities [7, 8]. Perhaps these contradictory findings can be partially explained by hunger. Al-Shawaf [33] reports that people in states of chronic or acute hunger have difficulties sustaining attention on food-irrelevant tasks, thereby compromising their general planning and problem-solving abilities. At the same time, however, hunger enhances memory of food stimuli [34] and the ability to solve food-acquisition problems [33].

Second, transitioning from childhood into adolescence or adulthood typically brings more opportunities for individuals to engage in community activities outside of the family home. And, compared to home, food is apt to be more readily available in community settings. As one parent noted “My 23-year-old is more independent now, and he has found a church down the street that feeds him.”

Convergent validity analyses of the FSZ confirmed the hypothesized relationship between the FSZ and hyperphagia. Regression analyses revealed that all but one FSZ factor was predictive of the total HQ-CT. Follow-up comparisons of FSZ factors across those with low, medium, or high HQ-CT scores revealed robust differences in the expected direction in all five FSZ factors. Importantly, age did not differ across HQ-CT tertiles, indicating that parents implement FSZ strategies in response to their child’s hyperphagic symptoms, not necessarily their age.

The Alerting Others and Community Supervision factor was not a significant predictor of hyperphagia. Even so, all items in this factor were frequently endorsed, especially in the youngest age group. It is possible that, given their child’s PWS diagnosis, families preemptively alert others about their child’s food issues as a baseline strategy. This widespread strategy remains in place, even as parents implement additional FSZ tactics in response to the changing needs of their individual with PWS.

Parental responses to the open-ended question offered poignant insights into both the logistics and stress of ensuring food safety. Several overarching messages emerged from their comments. First, given the high response rate to this question, parents were clearly motivated to explain their food safety practices.

Second, parental remarks highlighted that food safety is a life-long, round the clock pursuit, one that is especially challenging given the necessity for humans to eat and the omnipresence of food in social and community settings. And food safety requires constant vigilance. As one parent offered, “If the food is not secured and she gets it, then it is our fault. Not hers.”

Such constant vigilance, however, is highly stressful and associated with markedly high levels of caregiving burden. Indeed, levels of caregiving burden in PWS are high even as compared to parents of children with autism spectrum disorder or older caregivers of spouses with dementia [35]. Such parental burden relates to managing both their child’s hyperphagia and behavior problems, and has a profound, negative impact on their social and personal quality of life [35]. As one mother offered “My ENTIRE life is food security. 24/7, 24/7. We have NO life!”

Added to this burden is a counterintuitive psychological dilemma—the parental instinct to nurture and feed their hungry children juxtaposed with the reality that doing so could compromise their health and longevity. As one mother astutely remarked, “It is so hard to balance the psychology of being a parent, of wanting to feed a hungry child, with the dire medical repercussions of doing so.”

Finally, and as Table 7 depicted, there is no single “right” way of practicing food security, nor is there a cut-off score on the FSZ that indicates food safety has been met for all individuals with PWS. Instead, parents implement FSZ tactics that meet the individual and changing needs of their child while also considering what is feasible within the larger context of their family. Thus, some parents avoid attending restaurants or social gatherings, others find ways of navigating them. Some parents use locks and alarms, others do not.

Several study weaknesses should be noted. First, participants were generally White, well educated, and with relatively high annual incomes. Although SES was not related to the FSZ, future studies should include more economically or racially diverse participants. In this vein, one respondent offered that being homeless and in temporary housing made food safety nearly impossible. Second, test-retest reliability is typically assessed across shorter time frames than the 6-month interval used in the current study. Even so, mean FSZ scores at Time 1 and 2 were almost identical, and Intra-class correlations were strong. As well, we did not examine associations between the FSZ and family composition or marital status. Future research is needed on how, for example, siblings, grandparents or ex-spouses practice or perceive food safety.

Finally, we did not test our assumptions that the FSZ could help parents think less automatically and more deliberately as they evaluate their child’s hyperphagia. Instead, and as a first step, the study aimed to validate the FSZ for possible use in future research or clinical trials. If it is used, the FSZ should be administered prior to participants completing the HQ-CT. Clinical trial sponsors may also elect to use the FSZ as an exploratory outcome of family quality of life.

Despite these concerns, this study is the first to document and analyze food safety tactics in PWS. The 21-item FSZ emerged as a psychometrically robust measure of parental food safety tactics that holds promise for future phenotypic research, especially on family functioning, and clinical trials. In the meantime, the study also shines a much-needed light on the never-ending and extraordinary measures that parents use to ensure the health and wellbeing of their loved one with PWS.

## Supplementary Information

Supplementary Material 1.

## Data Availability

All data in aggregated form are contained in the manuscript and two Additional Files. Individual data are available upon reasonable request to the first author (ED) and require additional permission or approval from the Foundation for Prader-Willi Research (FPWR).
