# Supplementary material for: Validation of the Food Safe Zone questionnaire for families of individuals with Prader-Willi syndrome
Source: J Neurodev Disord. 2025 Feb 8;17:6. doi: 10.1186/s11689-024-09589-y (PMC11806870; doi:10.1186/s11689-024-09589-y)
Supplement: Supplementary file 1 — Supplementary Material 1. [file 11689_2024_9589_MOESM1_ESM.docx]

**Additional File Table 1.** FSZ item factor loadings, communalities, item means and relative frequencies from the follow-up study.

| **FSZ Factor Labels and Items** | **Factor Loading** | **Communalities** | **Item Means (SD)** | **% Most or All or the Time** | **% None or Some of the Time** |
| --- | --- | --- | --- | --- | --- |
| 1. **Alert Others, Supervision in the Community** |  |  |  |  |  |
| Alert others of child’s food issues to ensure they do not give child access to food. | .81 | .67 | 3.61 (.66) | 83.2% | 6.8% |
| Make sure adults involved with my child are aware of his/her food issues. | .80 | .68 | 3.81 (.52) | 97.5% | 2.5% |
| Make sure they are supervised while away from home. | .76 | .68 | 3.68 (.92) | 92.4% | 7.6% |
| Ensure child has no access to other people’s food at school, camp, or work. | .70 | .69 | 3.35 (.94) | 83.9% | 16.1% |
| Make a food plan for child prior to attending events, outings, restaurants* | .62 | .56 | 3.24  (.89) | 79.7% | 20.3% |
| 1. **Lock, Restrict Food Sources** |  |  |  |  |  |
| Lock up pantry or cabinets where food is kept | .84 | .82 | 2.56 (1.36) | 55.6% | 44.4% |
| Lock up refrigerator or freezer | .84 | .83 | 2.41 (1.39) | 48.3% | 51.7% |
| Ensure there is no food left on counter tops, tables, or other areas of access | .72 | .66 | 3.06 (1.00) | 74.6% | 25.4% |
| Lock up trash, compost, or recycling bins | .61 | .50 | 1.53 (.99) | 28.7% | 81.3% |
| Keep money or credit cards from child | .49 | .42 | 2.08 (1.33) | 38.1% | 61.9% |
| Use security features (alarm, camera) in home to monitor food access | .48 | .40 | 1.38 (.90) | 11.2% | 88.8% |
| 1. **Check for Food** |  |  |  |  |  |
| Check their belongings or bedroom for food, wrappers, or money | .89 | .86 | 1.64 (.99) | 18.7% | 81.3% |
| Check their person (pockets, pat down, shoes, etc.) for food, wrappers, or money | .84 | .82 | 1.45 (.82) | 14.4% | 85.6% |
| Check on child in bathroom to be sure he or she is not eating food | .77 | .72 | 1.47 (.78) | 12.7% | 87.5% |
| 1. **At Home Supervision, Meals** |  |  |  |  |  |
| Make sure child is busy while at home | .73 | .63 | 2.77  (.86) | 65.9% | 34.1% |
| Avoid eating in front of child unless they are also eating* | .69 | .55 | 2.86  (1.02) | 67.0% | 33.0% |
| Aware of where child is at home all the time (but may not be in sight of caregiver) | .62 | .68 | 3.33 (.76) | 89.0% | 11.0% |
| Supervise - always have eyes on at home | .53 | .52 | 2.84 (.99) | 67.8% | 32.2% |
| Make sure that meals are planned and on time | .42 | .41 | 3.19  (.81) | 85.6% | 14.4% |
| 1. **Avoid Food Settings** |  |  |  |  |  |
| Avoid taking child to restaurants | .78 | .69 | 1.86 (.88) | 29.7% | 69.3% |
| Avoid taking child to the grocery store | .74 | .64 | 1.88 (.92) | 29.9% | 70.1% |

**Additional File Table 2.** Mean FSZ factors scores between the large-scale and follow-up studies.

| **FSZ Factors** | **Large-Scale Study**  **M (SD)** | **Follow-Up Study**  **M (SD)** | **t’s*** |
| --- | --- | --- | --- |
| Alert Others, Supervision in the Community | 3.46 (.71) | 3.54 (.55) | 1.16 |
| Lock, Restrict Food Sources | 2.27 (.92) | 2.17 (.83) | 1.10 |
| Check for Food | 1.79 (.89) | 1.67 (.79) | 1.37 |
| At Home Supervision, Meals | 3.08 (.66) | 3.00 (.63) | 1.44 |
| Avoid Food Settings | 2.12 (.96) | 1.95 (.77) | 1.79 |

**Note:** *T tests were not significant.
